# Adrenocorticotropic Hormone-Producing Paraganglioma With Low Plasma ACTH Level: A Case Report and Review of the Literature

**DOI:** 10.3389/fendo.2019.00936

**Published:** 2020-01-21

**Authors:** Siyue Liu, Zhelong Liu, Fuqiong Chen, Weijie Xu, Gang Yuan

**Affiliations:** Department of Endocrinology, Tongji Hospital, Huazhong University of Science and Technology, Wuhan, China

**Keywords:** Cushing's syndrome, paraganglioma, adrenocorticotropic hormone, ectopic ACTH syndrome, immunohistochemistry, immunofluorescence

## Abstract

Ectopic adrenocorticotropic hormone (ACTH) syndrome caused by paraganglioma is extremely rare. It usually accompanied by high or normal plasma ACTH level. Here we described a male who presented with ectopic ACTH-producing paraganglioma and a low plasma ACTH level. Immunohistochemistry and immunofluorescence confirmed ACTH production in focal paraganglioma cells. This unusual case expanded the spectrum of ACTH-dependent Cushing's syndrome and revealed a potential mechanism of this unique clinical phenotype. Besides, the literature concerning ACTH-producing paraganglioma is reviewed.

## Background

Cushing's syndrome (CS) is a rare disorder with an incidence of five per million. CS is fatal unless appropriate treatment is provided; therefore, the early and correct diagnosis has important implications for patients (1). Most cases (about 80%) are caused by hypersecretion of adrenocorticotropic hormone (ACTH), 70% of which are primary pituitary diseases. Ten percent of CS cases are caused by ectopic ACTH production. The etiology of the remaining 20% of CS patients is not related to ACTH, but adrenal in origin (adrenal adenoma, cancer, or bilateral hyperplasia) (2). Paraganglioma and pheochromocytoma belong to a tumor of the paraganglion system; the former arises from the extra-adrenal regions and the latter from the adrenal medulla (3). Ectopic ACTH syndrome caused by paraganglioma is extremely rare. It usually accompanied by high or normal plasma ACTH level. Here we present an ectopic ACTH-dependent CS, caused by a paraganglioma. This is the first report of ectopic ACTH-producing paraganglioma with a low plasma ACTH level. This unusual case expanded the spectrum of ACTH-dependent CS and revealed a potential mechanism of this unique clinical phenotype. Besides, we review the literature concerning ACTH-producing paraganglioma.

## Case Presentation

**Figure 1:**
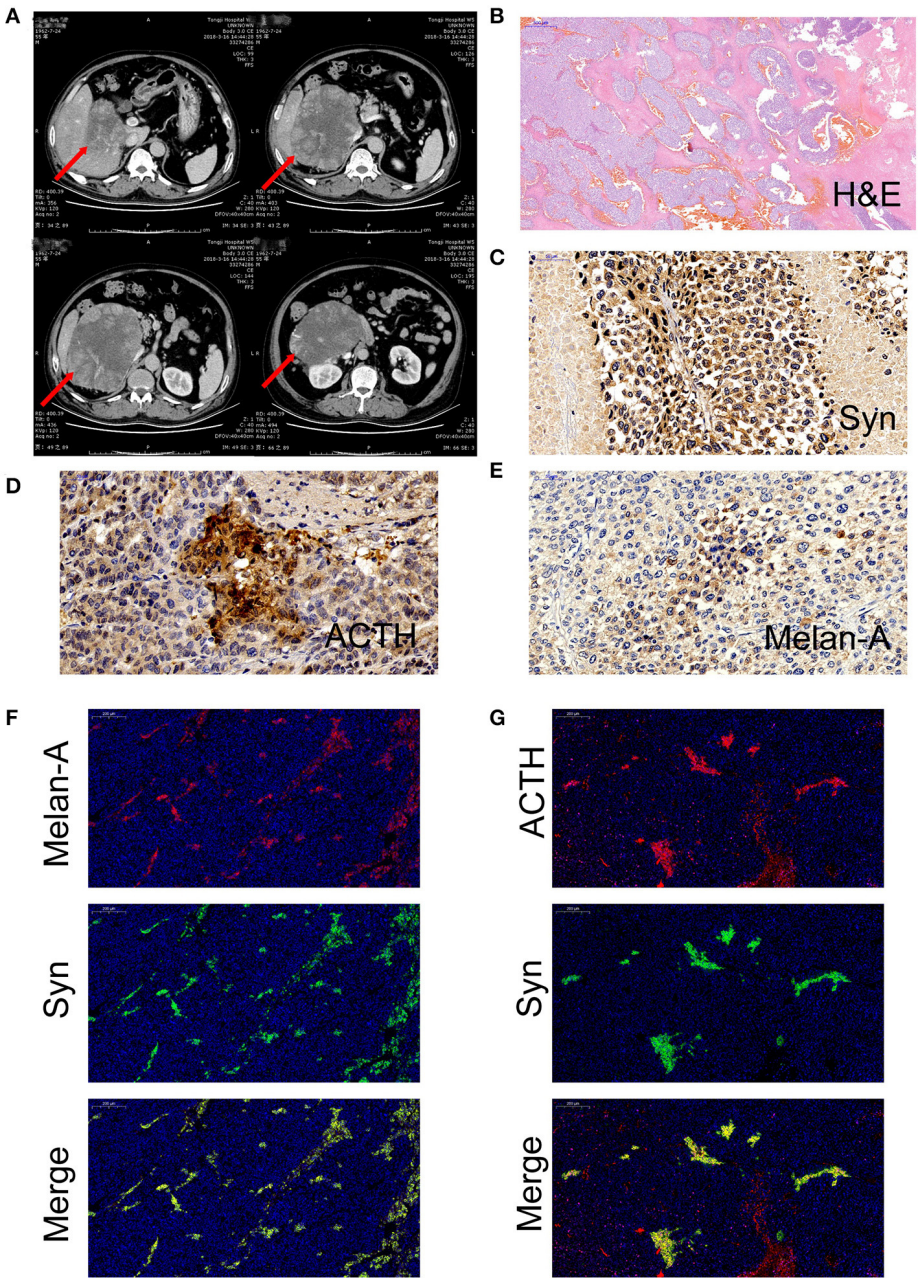
**(A)** Computed tomography of a large mass in Morison's pouch, measuring 17^*^12^*^12 cm (red arrow). **(B)** Hematoxylin and eosin (H&E) stain of paraffin embedded tumor tissue. **(C)** Immunohistochemistry with antibodies specific for synaptophysin (Syn) (Abcam, #ab32127, Cambridge, UK). **(D)** Immunohistochemistry with antibodies specific for adrenocorticotropic hormone (ACTH) (Abcam, #ab74976, Cambridge, UK). **(E)** Immunohistochemistry with antibodies specific for Melan-A (Abcam, #ab210546, Cambridge, UK). **(F)** Double immunofluorescence staining for synaptophysin (green) and Melan-A (red). **(G)** Double immunofluorescence staining for synaptophysin (green) and ACTH (red).

A 55-year-old man presented with a 2-month history of severe hypertension (220/160 mmHg). Blood pressure was maintained at 150/100 mmHg with benzenesulfonate levamlodipine 5 mg treatment. There was no apparent headache, palpitation, and hyperhidrosis. He also suffered from persistent distended upper abdominal pain and fatigue for 2 weeks. There was no family history of Cushing's syndrome or pheochromocytoma. Physical examination revealed a blood pressure of 148/102 mmHg, a heart rate of 98 beats/min. He showed no cushingoid features such as hyperpigmentation, muscle weakness of the limbs, moon face, or buffalo hump. Laboratory examination showed the presence of slight hypokalemia (Table 1). The serum level of cortisol was elevated, yet the ACTH level was decreased (Table 2). There was no suppression after 2-day 2-mg dexamethasone administration (Table 2). There was no elevation of renin, aldosterone, urinary metanephrine, and normetanephrine levels (Table 2). Subsequent analysis of 24-h urinary metanephrine, normetanephrine, catecholamines, and vanillylmandelic acid, as well as of blood catecholamines, showed no elevated levels. B-scan ultrasonography, computed tomography (CT) scan, and enhanced scans presented a large mass in Morison's pouch, measuring 17^*^12^*^12 cm, possibly derived from the right adrenal gland (Figure 1). The images showed no evidence of left adrenal hypertrophy, respectively. According to these findings, our clinical diagnosis was Cushing's syndrome with a retroperitoneal mass. Alpha-blocker and calcium channel blocker were added, and he underwent an exploratory laparotomy, retroperitoneal tumor resection, and right adrenalectomy. His right adrenal gland is compressed and atrophic, carrying no tumor cells, and no hyperplasia was evident. The resected tumor was diagnosed as the ACTH-secreting paraganglioma in the pathological examination. Histological features were typical of paraganglioma, including chief cells arranged in nests, alveolar-like, and stereo-like structures and surrounded by sustentacular cells partly or entirely (a Zellballen pattern). Immunohistochemical analysis revealed scattered and focally positive for synaptophysin in tumor cells and S-100 positivity for sustentacular cells, which are characteristics of paraganglioma. Additional immunohistochemistry and double immunofluorescence technology revealed positive immunostaining for ACTH, and also for synaptophysin, proving that ACTH secretion indeed was derived from paraganglioma cells. Furthermore, immunofluorescence histochemical double staining was positive for both Melan-A and synaptophysin in focal tumor cells, indicating that these ACTH-secreting tumor cells might secrete cortisol as well (Figure 1). All of these findings confirmed the diagnosis of ACTH-secreting paraganglioma and Cushing's syndrome. After surgery, his hypertension and the symptoms of abdominal pain and fatigue improved, and the hydrocortisone supplementation slowly tapered. At the last follow-up 1.5 years later, his blood pressure and heart rate became normal (128/80 mmHg and 78 bpm); his plasma ACTH level increased, and the cortisol level dropped to the normal range. Hypokalemia was improved (Table 2).

**Table 1:** Baseline laboratory values of the patient.

**Parameter**	**On admission**	**Postoperative**	**Reference range**
WBC	10.1	8.3	5.2–11.4 10^3^/μL
Hg	14	14	12–16 g/dL
Plt	246	284	130–400 10^3^/μL
Neutrophil	7.57	5.84	1.9–8 10^3^/μL
Eosinophil	0.1	0.0	0-0.8 10^3^/μL
Lymphocyte	2.5	2.4	0.9–5.2 10^3^/μL
Glucose	85	78	70–100 mg/dL
Na^+^	140.6	142.8	136–145 mmol/L
K^+^	3.4	4.7	3.5–5.1 mmol/L

*WBC, white blood cell; Hg, hemoglobin; Plt, platelet; Na^+^, sodium; K^+^, potassium*.

**Table 2:** Hormone profiles and dexamethasone suppression test.

**Parameter**	**On admission**	**Postoperative**	**Reference range**
ACTH	1.0	76.3	7.2–63.3 pg/mL
Cortisol	18.60	10.90	6.02–18.4 μg/dL
Aldosterone	27.1	30.2	0–353.0 pg/mL
Plasma renin activity	13	14	4.4–46.1 μIU/mL
Urinary metanephrine	145.28	150.82	38–266 μg/24 h
Urinary normetanephrine	116.54	114.86	27–561 μg/24 h
Plasma metanephrine	<0.07	<0.07	≤ 0.21 nmol/L
Plasma normetanephrine	<0.06	<0.06	≤ 0.59 nmol/L
Urinary epinephrine	1.1	2.5	0–14 μg/24 h
Urinary norepinephrine	38.5	27.9	1–100 μg/24 h
Urinary dopamine	167.83	108.57	18–504 μg/24 h
Urinary vanillylmandelic acid	16.8	21.4	0–41.28 μmol//24 h
Urinary homovanillis acid	15.14	8.66	0–41.86 μmol//24 h
TSH	0.919	ND	0.27–4.2 μIU/mL
fT3	2.11	ND	2.0–4.4 pg/mL
fT4	11.56	ND	9.32–17.09 ng/L
**Dexamethasone suppression test before surgical operation**			
Dexamethasone	Basal	2 mg	
Cortisol (μg/dL)	18.5	19.10	

*ACTH, adrenocorticotropin; TSH, thyroid stimulating hormone; fT3, free triiodothyronine; fT4, free thyroxine; ND, no data*.

## Discussion

This case represents a very rare cause of ectopic CS caused by an ACTH-producing paraganglioma and illustrates the diagnostic challenges of ACTH-dependent CS. This is the first report of ectopic ACTH-producing paraganglioma with a low plasma ACTH level. It demonstrates that the relative contributions of clinical, biochemical, and radiological clues in establishing the correct underlying cause of CS may differ considerably between Cushing's disease and Cushing's syndrome due to ectopic ACTH production.

In about 90% of cases, tumors arising from chromaffin cells are located in the adrenal medulla and are commonly termed pheochromocytomas, whereas, in 10% of cases, tumors are extra-adrenal and are termed paragangliomas (3). Hormonal and immunobiological studies suggested that our patient suffered from functional paragangliomas with ACTH producing. Only 15 cases of ectopic ACTH caused by paraganglioma were reported (Table 3) (4–18). The tumors were located in mediastinum in 4 cases, paranasal sinuses in 5 cases and retroperitoneal in 3 cases. Two cases were malignant. The ages of all the patients ranged from 12–70 years old; 11 of them were female. There were 14 cases of hypertension and 12 cases of hyperglycemia. Hypokalemia occurred in 9 cases. Consistent with previous studies, only three patients experienced excessive catecholamine excretion. Although paraganglioma originated from chromaffin cells, only about 16.9% of the patients showed an increase in catecholamine, which may be due to the fact that the tumor body of paraganglioma is usually large, and catecholamine may breakdown within the tumor body and fail to be released into the blood. It may also be that some paragangliomas do not produce catecholamine at all. On the other hand, about 95% of paraganglioma patients with catecholamine hypersecretion presented with hypertension, while only 33.5% of paraganglioma patients without elevated catecholamine had hypertension (19). For ACTH producing paraganglioma patients, although only 3 of 15 patients showed catecholamine hypersecretion, 14 patients had hypertension, indicating that the hypertension that occurred in these patients could be derived from Cushing's syndrome.

**Table 3:** Clinical characteristics of patients with ACTH-producing paraganglioma published in the literature.

**Authors**	**Age/gender**	**AM Cortisol (μg/dl)**	**ACTH (pg/ml)**	**Hypertension**	**Hyperglycemia**	**Hypokalemia**	**Catecholamine excess**	**Location**	**Clinical outcome**
Apple et al. (4)	50/F	52	167	+	+	ND	ND	Right nasal sinuses	Recovery
Kitahara et al. (5)	12/F	107.1	13.6	+	+	−	+	Lung;	Died; malignant paraganglioma
Park et al. (6)	51/F	59	278	+	+	+	−	Anterior mediastinum	Died of mediastinitis
Lieberum et al. (7)	64/M	High, ND	95.6	+	+	+	−	Paranasal sinus	Recovery
Dahir et al. (8)	39/F	30.6	73.0	+	ND	ND	−	Mediastinum	Recovery
Otsuka et al. (9)	55/F	76.5	318.4	+	+	+	+	Retroperitoneum	Recovery
Willenberg et al. (10)	61/F	176.0	1078	+	+	+	+	Retroperitoneum	Died of pulmonary bleeding 6 months after operation
Palau et al. (11)	55/M	High, ND	High, ND	ND	ND	ND	ND	Mediastinum	Recovery
Fohr et al. (12)	23/M	38	287	+	+	ND	ND	Anterior mediastinum	Recovery
Thomas et al. (13)	70/F	74.4	273.0	+	+	+	−	Left paranasal sinus	Recovery
Serra et al. (14)	68/F	98.7	317.0	+	+	+	ND	Right nasal sinuses	Recovery
Chen et al. (15)	53/F	89.6	432.4	+	+	+	−	Retroperitoneum	Recovery
Kumar et al. (16)	46/F	94.6	312.0	+	+	ND	ND	Right nasal sinuses	Recovery
Tutal et al. (17)	40/F	61.1	679	+	+	+	−	Left kidney	Recovery
Li et al. (18)	39/M	37.68	151.50	+	ND	+	−	Thymus	Recovery
Present case	55/M	18.60	1.0	+	−	+	−	Retroperitoneum	Recovery

*All are paraganglioma proved by immunohistochemistry. ACTH, adrenocorticotropin; F, female; M, male; ND, not documented*.

In general, most patients with ACTH-secreting paraganglioma presented with significantly elevated plasma ACTH levels. In the literature, only one of 15 cases presented with normal plasma ACTH level and small (pg) amounts of ACTH in tumor extract. Interestingly, the plasma ACTH level was suppressed in our patient. Louiset et al. described a complex paracrine regulation of cortisol secretion resulting from the unexpected expression of ACTH in clusters of steroidogenic cells in bilateral macronodular adrenal hyperplasia tissues. Cortisol secretion by the adrenals in patients with macronodular hyperplasia and Cushing's syndrome appears to be regulated by corticotropin, which is produced by a subpopulation of steroidogenic cells in the hyperplastic adrenals (20). Similarly, in our case, ACTH was immunohistochemically detectable in focal tumor cells, as well as Melan-A and synaptophysin. Synaptophysin, an integral membrane protein of small synaptic vesicles in the brain and endocrine cells, is abundant in neuroendocrine cells and tumor tissues with neuroendocrine function. Synaptophysin is mainly expressed in adrenal medulla, pheochromocytoma, and paraganglioma (21, 22). On the other hand, Melan-A is present in the cytoplasm of epithelial cells and steroid hormone-secreting cells. It is often expressed in melanoma and adrenal cortex (23, 24). In conclusion, these results indicate that the paraganglioma in our case indeed produced ACTH and cortisol. The mildly elevated concentrations of plasma cortisol suggest that the tumor cells produced cortisol and secreted it. As for ACTH, biochemistry failed to demonstrate its excess in the blood. These results may suggest that the tumor cells produced a small amount of ACTH, which stimulates the synthesis of cortisol by cortisol-producing cells in the tumor through an autocrine or paracrine pattern. Excessive cortisol would then inhibit the secretion of ACTH in the pituitary as a negative feedback, accounting for the suppressed plasma level of ACTH. After surgery, the patient's plasma ACTH level increased and the cortisol level dropped to the normal range, confirming the above speculation. This is the first and unique report of ectopic ACTH-producing paraganglioma with a low plasma ACTH level.

The hypersecretion of cortisol may result in hyperglycemia and suppression of the immune system. Thus, before tumor resection, patients are commonly susceptible to infections. In the literature, seven out of 15 patients presented with infections, and one patient died of mediastinitis and pneumonia. In our case, the patient had pneumonia, and his pneumonia did not improve until the paraganglioma was resected.

We did not perform a high dose dexamethasone suppression test to distinguish orthotopic and ectopic ACTH secretion, given its relatively low diagnostic value in this diagnostic setting (the retroperitoneal tumor must be treated regardless of test results) and its risk of hypertensive crisis (25).

CS can also manifest as metabolic syndromes, such as hypertension, hyperglycemia, and hypokalemia. It is challenging to detect the ACTH source in CS. In such settings, biochemical and imaging assessments can prove useful. In the present case, we were confronted by an extremely rare ACTH-producing retroperitoneal paraganglioma with a low plasma ACTH level. Cortisol secretion by the paraganglioma in the patient with CS appears to be regulated by ACTH, which is produced by these steroidogenic tumor cells in the paraganglioma. Surgical resection is the preferred and definitive treatment. This unusual case expanded the spectrum of ACTH-dependent CS and revealed a potential mechanism of this unique clinical phenotype.

## Data Availability Statement

All datasets generated for this study are included in the article.

## Ethics Statement

The studies involving human participants were reviewed and approved by Ethics Committee, Tongji Hospital of Tongji Medical College, Huazhong University of Science and Technology. The patients/participants provided their written informed consent to participate in this study. Written informed consent was obtained from the individual(s) for the publication of any potentially identifiable images or data included in this article.

## Author Contributions

GY and WX conceived and designed the study. SL and ZL performed the experiments and wrote the paper. FC and GY reviewed and edited the manuscript. All authors read and approved the manuscript.

### Conflict of Interest

The authors declare that the research was conducted in the absence of any commercial or financial relationships that could be construed as a potential conflict of interest.
